# Proteomics and Lipidomics Investigations to Decipher the Behavior of *Willaertia magna* C2c Maky According to Different Culture Modes

**DOI:** 10.3390/microorganisms8111791

**Published:** 2020-11-16

**Authors:** Issam Hasni, Nicholas Armstrong, Philippe Decloquement, Said Azza, Anthony Fontanini, Olivier Abbe, Amina Cherif Louazani, Sandrine Demanèche, Eric Chabrière, Philippe Colson, Bernard La Scola

**Affiliations:** 1 Aix-Marseille Université UM63, Faculté des Sciences Médicales et Paramédicales secteur Timone, Institut de Recherche Pour le Développement IRD 198, Assistance Publique—Hôpitaux de Marseille (AP-HM), 13385 Marseille, France; issemhasni@gmail.com (I.H.); nicholas.armstrong@univ-amu.fr (N.A.); Philippe.DECLOQUEMENT@univ-amu.fr (P.D.); said.azza@univ-amu.fr (S.A.); cheriflamina@gmail.com (A.C.L.); eric.chabriere@univ-amu.fr (E.C.); philippe.colson@univ-amu.fr (P.C.); 2 R&D Department, Amoéba, 69680 Chassieu, France; o.abbe@amoeba-nature.com (O.A.); s.demaneche@amoeba-nature.com (S.D.); 3 Institut Hospitalo-Universitaire (IHU), Microbes, Evolution, Phylogeny and Infection (MEΦI)—Méditerranée Infection, 13005 Marseille, France; fontanini.anthony@gmail.com

**Keywords:** *Willaertia magna* C2c Maky, amoebas, culture, proteomics, lipidomics, metabolism

## Abstract

*Willaertia magna* C2c Maky is a free-living amoeba that has demonstrated its ability to inhibit the intracellular multiplication of some *Legionella pneumophila* strains, which are pathogenic bacteria inhabiting the aquatic environment. The Amoeba, an industry involved in the treatment of microbiological risk in the water and plant protection sectors, has developed a natural biocide based on the property of *W. magna* to manage the proliferation of the pathogen in cooling towers. In axenic liquid medium, amoebas are usually cultivated in adhesion on culture flask. However, we implemented a liquid culture in suspension using bioreactors in order to produce large quantities of *W. magna*. In order to investigate the culture condition effects on *W. magna*, we conducted a study based on microscopic, proteomics and lipidomics analyzes. According to the culture condition, amoeba exhibited two different phenotypes. The differential proteomics study showed that amoebas seemed to promote the lipid metabolism pathway in suspension culture, whereas we observed an upregulation of the carbohydrate pathway in adherent culture. Furthermore, we observed an over-regulation of proteins related to the cytoskeleton for *W. magna* cells grown in adhesion. Regarding the lipid analysis, suspension and adhesion cell growth showed comparable lipid class compositions. However, the differential lipid analysis revealed differences that confirmed cell phenotype differences observed by microscopy and predicted by proteomics. Overall, this study provides us with a better insight into the biology and molecular processes of *W. magna* in different culture lifestyles.

## 1. Introduction

*Willaertia magna* is a free-living amoeba (FLA) belonging to the class *Heterolobosea* and family *Vahlkampfiidae* [1,2]. This FLA is found in natural and artificial environments, such as humid soil, bovine feces, composts, thermal waters, and fresh water sediments [3,4,5]. *W. magna* life cycle is composed of three stages switching between trophozoite form, flagellate form and cyst form [1]. The trophozoites stage is characterized by a large form containing one or several nuclei, food and contractile vacuoles, and mitochondria. The phagotrophic trophozoites feed on bacteria and can grow within a range of 22 °C to 44 °C [4]. The amoeboid form can temporary transform into ameboflagellate under certain conditions [6]. To resist unfavorable conditions, the trophozoite has the capacity to transform into large cysts containing pores in the cell wall [4]. Although *W. magna* is phylogenetically close to *Naegleria fowleri*, a virulent amoeba causing primary amoebic meningoencephalitis (PAM) in humans, in vivo and in silico experiments have demonstrated the non-pathogenicity of *W. magna* [4,7,8]. The analysis of the *W. magna* draft genome revealed a DNA length of 36.5 megabases and less than 19,000 genes [8].

*Legionella pneumophila* is a Gram-negative bacterium causing legionellosis, a severe and potentially fatal pneumonia in humans [9]. Although *L. pneumophila* is ubiquitous in natural water environment [10], the numerous cases of legionellosis are mainly due to the inhalation or respiration of pathogenic bacteria present in engineering water systems, such as cooling towers where legionella finds suitable growing condition [11,12,13]. Furthermore, the co-existence of FLAs and *L. pneumophila* complicates the monitoring of the latter in cooling tower waters [14,15]. *L. pneumophila* strains have the capacity to infect and invade a wide range of amoebas, including *Vermamoeba*, *Acanthamoeba,* and *Naegleria* species [12,16,17,18]. Furthermore, amoeba cysts provide to *L. pneumophila* a protection against harsh conditions and chemical treatments [15]. Regarding *W. magna* strain C2c Maky, it has been shown to be capable of phagocyting and inhibiting the intracellular growth of certain human pathogenic strains of *L. pneumophila* [19,20].

This finding led the Amoeba company, an industry involved in the sanitation of water in cooling towers and in the treatment of plant protection, to develop a natural biocide to monitor and prevent *L. pneumophila* proliferation water in cooling towers as an alternative to chemical biocides that are not completely efficient [21]. However, the treatment of water in cooling towers by this mean requires large quantities of *W. magna*. The traditional culture of amoebas is performed on agar plate or liquid support in xenic or axenic medium. Nevertheless, these culture methods do not allow to obtain large amounts of amoebas in a short time [22]. Axenic mass culture of *Acanthamoeba castellanii* in bioreactors has already been shown to improve the production of this amoeba [23]. In a previous study, Weekers et al. have carried out the axenic culture of *A. castellanii* in a fermenter and analyzed the behavior of this amoeba by investigating different parameters of the growth process, such as glucose consumption, amoeba concentration or respiration [24]. In another study, the behavior of *Dictyostelium discoideum* during a culture in suspension was investigated [22].

According to the culture conditions, the organisms need to regulate their metabolism and to mediate molecular responses in order to adapt to various environments [25]. Indeed, variations in gene expression and protein synthesis lead to different phenotypes and allow adaptation to distinct environments. Furthermore, it is known that the culture condition has an effect on the morphology, proteome and lipid composition of cellular organisms [25,26,27]. So far, the behavior of amoebas grown under different growing conditions remains unclear, especially for suspension and adhesion cell growth [28,29]. Over the past few decades, multi-omics approaches, including genomics, transcriptomics, proteomics, or lipidomics analyses have become essential tools for exploring the biology, behavior, molecular mechanisms, or metabolism of organisms [30,31,32]. However, these methods have not yet been widely applied to study FLAs. Indeed, only a few studies based on genomics, transcriptomics and proteomics are described in the literature [8,33,34,35,36,37,38,39,40,41,42].

In a previous study, we explored the behavior of *W. magna* C2c Maky in suspension culture by transcriptomics and proteomics analyzes [33]. To decipher the effect of culture conditions on *W. magna* C2c Maky, we explored the morphologic differences of *W. magna* growing in two culture modes by microscopic analysis. Then, we investigated the differential regulation of proteins for *W. magna* cultivated according to two different culture methods. Finally, an analysis of lipid classes was performed to further decipher the *W. magna* phenotype associated with the culture methods.

## 2. Materials and Methods

### 2.1. Culture of Willaertia magna C2c Maky in Adhesion

Culture of *Willaertia magna* C2c Maky (ATCC PTA-7824) was performed at 30 °C (Thermo Fisher Scientific, Illkirch, France) using 175 cm² culture flasks in SCGYEM medium [43]. When the trophozoite formed a monolayer, the amoebas were detached by tapping the culture flasks and harvested by centrifugation at 2000× *g* during 10 min, followed by three steps of washing using page’s amoeba saline (PAS) medium (2 mM NaCl, 16 μM MgSO_4_, 27.2 μM CaCl_2_, 1 mM Na_2_HPO_4_, 1 mM KH_2_PO_4_). Amoeba quantification was performed using a KOVA^®^ slide cell counting chamber.

### 2.2. Culture of Willaertia magna C2c Maky in Suspension

*W. magna* C2c Maky was cultivated in a 10-L bioreactor (GPC, La Rochelle, France; [44]) in modified SCGYEM medium without fetal calf serum (pH = 7), as previously described [37]. To perform morphologic, proteomics, and lipidomics analyses, the amoeba cells were harvested from bioreactor in a volume of 50 mL, and centrifugated at 2000× *g* for 10 min and washed in three steps using PAS medium. Amoeba quantification was performed using a KOVA^®^ slide cell counting chamber.

### 2.3. Protein Extraction

Amoebas were harvested from three different bioreactors or three different flasks in order to perform a proteomics study on three biological replicates. For each condition, we put 40 mL of amoebas at 10^6^ amoebas/mL in 50 mL falcon tubes (Dutscher SAS, Brumath, France) in medium culture. The amoebas were rinsed with PAS and centrifugated at 2000× *g* for 10 min. Four sets of samples from each *W. magna* of different condition culture were prepared for label-free Nano-LC-MS/MS analysis. Each sample was solubilized as previously described [45]. Briefly, samples were suspended in 200 μL of lysis buffer (100 mM Tris-HCl, pH 8.0, supplemented with 2% (wt/vol) sodium dodecyl sulfate and 100 mM dithiothreitol) followed by 5 min of heating at 95 °C. After a 3-min sonication at 20 W, the insoluble fraction was removed by centrifugation (12,000× *g*, 20 min) and soluble proteins were precipitated using a PlusOne 2-D cleanup kit (GE Healthcare) to remove SDS. The final pellet was resuspended in 200 µL of solubilization buffer (Urea 8M, Thiourea 2M, 100 mM NaCl, 25 mM Tris, pH 8.2) and dialyzed twice against 1 L of 50 mM ammonium bicarbonate pH 7.4, Urea 1M (4 h and overnight) using Slide-ALyzer Dialysis Cassettes 2K MWCO (Pierce Biotechnology, 122 Rockford, IL, USA). Dialyzed fractions were collected, and proteins were quantified by Bradford assay using Coomassie (Biorad). The dialyzed fraction was used as template for global proteomic analysis. Briefly, 50 µg of total soluble proteins were reduced with 10 mM dithiothreitol (Euromedex, Souffelweyersheim, France) for one hour at 30 °C, and then alkylated with 20 mM iodoacetamide (Sigma, Saint-Quentin Fallavier, France) for one hour in the dark. Protein digestion was performed by adding 2 µg of sequencing-grade trypsin solution (Promega, Charbonnières, France) to alkylated proteins and incubated overnight at 37 °C. The digested sample was then desalted using Pierce Detergent Removal Spin Columns (Thermo Fisher Scientific, Illkirch, France) and analyzed by mass spectrometry, as described below. For the three biological replicates of amoebas cultivated in adhesion, we obtained protein concentrations of 1.9, 2.5, and 1.1 (µg/µL) respectively. For amoebas cultivated in suspension, we obtained protein concentrations of 4.4, 4.7, and 4.7 (µg/µL). Table S1 presents the complete results obtained from protein extraction.

### 2.4. Label-Free Quantitative Nano-LC-MS/MS Proteomic Analysis

Protein digests were first separated by Ultra Performance Liquid Chromatography (UPLC) using the NanoAcquity UPLC System (Waters, Milford, CT, USA) connected to a Synapt G2Si Q-TOF ion mobility hybrid mass spectrometer (Waters). The chromatographic system was used in 1D configuration with an analytical column (ACQUITY UPLC M-Class peptide CSH C18 Column, 130Å 1.7 µm, 75 µm × 100 mm, Waters) after a trapping column (ACQUITY UPLC M-Class Symmetry C18 Trap Column, 100Å 5 µm 2G V/M, 180 µm × 20 mm, Waters). Eluted peptides were then separated using a 100 min gradient (300 nL·min^−1^; 0.5 to 40% acetonitrile–0.1% formic acid). Data-independent MS/MS analysis was performed with the ion mobility feature (HDMSe method). The parameters of ion source were as follows: Capillary voltage at 3 kV, sampling cone voltage at 40 V, ion source temperature at 90 °C, cone gas flow at 50 L·h^−1^. Transfer collision low energy was set to 5 V and trap collision low energy was set to 4 V. The high energy ramp was applied from 4 V to 5 V for the trap collision and from 19 V to 45 V for the transfer collision enabling fragmentation of the ions after the ion mobility cell and before the time-of-flight (TOF) MS. Each sample was injected in triplicate.

The acquired files were imported into Progenesis QI software Version 2.0 (Nonlinear Dynamics, Newcastle, UK) for label-free quantification analysis. The data were automatically aligned against *W. magna* proteins (https://www.mediterranee-infection.com/acces-ressources/donnees-pour-articles/willaertia-magna-c2c-maky/) and normalized. Processing parameters were 150 counts for the low energy threshold and 30 counts for the elevated energy threshold. The database used was the same as the one described above. Search tolerance parameters were peptide and fragment tolerance, 15 ppm, FDR < 1%; Minimum Ion matching requirements were three fragments per peptide, seven fragments per protein and two peptides per protein. The enzyme specificity was trypsin allowing 1 missed cleavage. The accepted modifications were carbamidomethyl of cysteine (fixed), oxidation of methionine (variable), carbamylation of lysine and N-terminal (variable), and deamidation (variable) of asparagine and glutamine. Protein normalization was performed according to the relative quantitation using non-conflicting peptides. To determine the significance of changes between samples, a significant ANOVA (*p*-value < 0.001) and a fold change greater than 2 were used as thresholds to define differently regulated proteins (DRPs). The results obtained from proteomics analysis are detailed in Table S2.

### 2.5. Analysis on Biological Function of Differentially Regulated Proteins

To assign the biological functions of DRPs, we proceeded to homology searches for the proteins in public protein databases. First, the protein sequences were searched by BLASTp against the NCBI non redundant protein sequence database with an E-value cut-off of 1 × 10^4^ [46]. We mapped the protein sequences against the Cluster of Orthologous Group of proteins (COG) and Gene Ontology (GO) databases using EggNOG [47,48] with diamond as mapping mode. The visualization of GO annotation was performed using WEGO online software [49]. In a second time, we compared the protein sequences against the Kyoto Encyclopedia of Genes and Genomes Pathway (KEGG; http://www.genome.jp/kegg) using BLASTKoala online [50]. Conserved domain database (CDD) and InterPro were used for the identification of conserved domains in the sequence of DRPs [51,52,53].

### 2.6. Quantitative Real-Time Reverse-Transcription-Polymerase Chain Reaction (qRT-PCR)

In order to validate the DRP results, 10 genes encoding DRPs were selected to quantify their expression by real-time quantitative reverse transcription PCR (qRT-PCR) using SYBR Green (Roche, Meylan, France) and 1 gene encoding a histone protein was used as internal control. To choose the internal control, we identified the set of proteins whose regulation was not affected by culture conditions. Among these proteins, we selected a panel of candidate genes. We quantified expression level of these candidate genes under two different culture conditions (suspension and adhesion). We selected the most stable gene among the set of reference candidate genes tested. RNAs from *W. magna* in each culture condition were extracted using the RNeasy Mini Kit (Qiagen Corp., Hilden, Germany) following the manufacturer’s protocol. Total RNA was eluted in a 50 μL volume of RNase-free water. RNaseOUT (Thermo Fisher Scientific, Illkirch, France) was added to the eluate to prevent RNA degradation. DNA digestion was performed using Turbo DNase (Life Technologies, Carlsbad, USA) at 37 °C for 4 h. The design of primer for each gene was performed using the Primer3 website (http://frodo.wi.mit.edu/primer3/). Primers are listed in Table S3. Extracted RNAs were reverse transcribed into cDNA using random primers with the SuperScript VILO Synthesis Kit (Invitrogen, Cergy-Pontoise, France). The synthesized cDNAs were purified with the Agencourt AMPure XP system (Beckman Coulter Inc., Brea, CA, USA). Quantitative PCR was carried out in a CFX96 real time system (Biorad) using the following cycling conditions: 95 °C for 5 min, followed by 45 cycles of 95 °C for 10 s, annealing at 60 °C for 30 s and elongation at 72 °C for 30 s. As in Li et al.’s study, the relative expression (fold change) of each gene was calculated with the 2−ΔΔCt method and statistics were generated using Student’s *t*-test [54,55]. All experiments were performed with three biological replicates.

### 2.7. Analysis of Lipid Classes by Hydrophilic Interaction Liquid Chromatography-Mass Spectrometry (HILIC-MS)

For each condition, triplicate amoeba cultures were centrifuged at 6000× *g* during 10 min in order to collect humid cell pellets. Total lipids were extracted according to the Bligh and Dyer protocol [56]. Chloroform extracts were then dried under a stream of nitrogen and reconstituted in chloroform/methanol 50% (*v*:*v*) at a final concentration of 6 mg of lipid content per 100 µL. Lipid samples were diluted 100 times into methanol before injection (5 µL) onto a HILIC column (BEH HILIC, 2.1 × 100 mm, 1.7 µm, Waters, Guyancourt, France). Lipids were eluted from the column using a composition gradient of the following solvents: A = 5% water/95% acetonitrile, B = 50% water/50% acetonitrile, both at 10 mM ammonium acetate pH8 as previously described [57]. Lipids were ionized in the positive and negative ionization modes using a Z-spray source (2.8 kV, 35V for ESI+ and 1.9 kV 40 V for ESI-). Ions were then monitored using an HD-MS method including ion mobility and single stage MS scans (ranged from 50 to 2000 *m*/*z*, 0.1 s per scan, lockmass calibration using Leucine Enkephaline). Multivariate statistics were performed for each ionization mode on all detected ion components using the UNIFI and EZ info software (with normalization of data) [58]. A PLS-DA model was calculated from the principal component analysis (PCA) in order to select form S-Plot (>97%) and VIP (>1) plots, the most discriminating markers for both culture conditions. Lipid classes were attributed according to the retention times of an injected standard (Splash Lipidomix, Avanti Polar Lipids, Alabaster, AL, USA). Markers masses were then searched with the COMP DB LipidMAPS database with a delta window of 0.005 *m*/*z* and all enabled chains. The database lipid charge was checked versus the ion component extracted from the MS raw data.

### 2.8. Fatty Acid Methyl Ester (FAME) Analysis by Gas Chromatography/Mass Spectrometry

Cellular fatty acid methyl ester (FAME) analysis was performed by GC/MS, as previously described [59,60]. Fatty acid methyl esters were prepared as described by M. Sasser [61]. Briefly, fatty acid methyl esters were analyzed by gas chromatography/mass spectrometry (GC/MS). Compounds were separated using an Elite 5-MS column and monitored by mass spectrometry (Clarus 500—SQ 8 S, Perkin Elmer, Courtaboeuf, France). Spectral database search was performed using MS Search 2.0 operated with the Standard Reference Database 1A (NIST, Gaithersburg, MD, USA) and the FAMEs mass spectral database (Wiley, Chichester, UK).

### 2.9. Electron Microscopy

For scanning electronic microscopy, a suspension of *W. magna* in trophozoite forms (for the amoebas cultivated in suspension and in adhesion) form was immersed into a 2.5% glutaraldehyde fixative solution. A drop of the suspension was deposited on a slide, then gently washed with water, air-dried and examined under the Emission Scanning Electron Microscope SU5000 (approximately 33 cm wide by 60 cm tall, Hitachi, Japan).

## 3. Results

### 3.1. Phenotype of Willaertia magna Cultivated under Two Different Conditions

**Figure 1:**
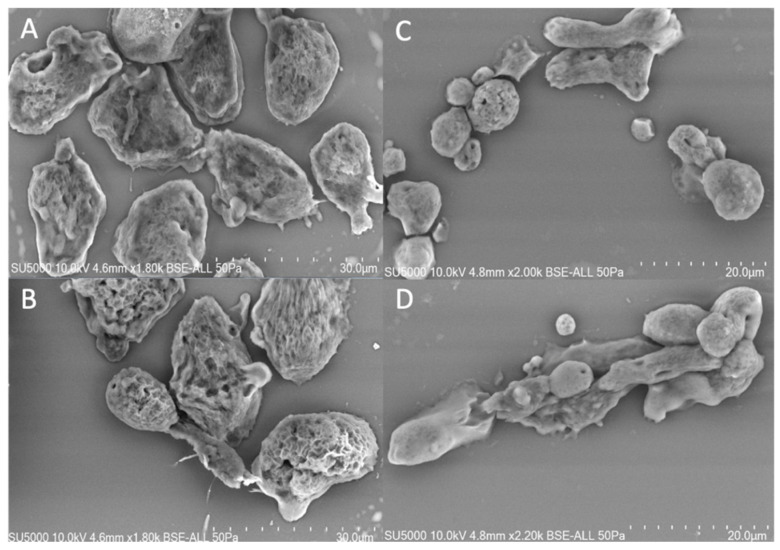
Morphology of *Willaertia magna* cultivated under two different culture conditions. In (**A**,**B**): *W. magna* is cultivated in adhesion on cell culture flask. In (**C**,**D**): *W. magna* is cultivated in suspension in bioreactor. Pictures were obtained with emission Scanning Electron Microscope SU5000 (Hitachi, Japan). Bar scales are represented on picture.

The adherent amoebas exhibited an irregular shape that constantly changed giving rise to the presence of several pseudopods (Figure 1A,B). The suspended amoebas exhibited a more regular shape with a more rounded morphology and exhibited much fewer pseudopods (Figure 1C,D). The length of the amoebas cultivated under two different conditions exhibited approximately similar size, with length of 21 µm (+/−3.34) and 19.5 µm (+/−4.50) for the amoeba grown in adhesion and suspension, respectively (Table S4).

However, the amoeba in adhesion (14.2 µm) is approximately 3 times width than the cultivated amoebas in bioreactor (5.2 µm). We observed the presence of pseudopodia for amoebas in adhesion, which is characteristic of the active movement of the amoebas.

### 3.2. Identification of Differentially Regulated Proteins

A total of 804 non-redundant proteins were identified using UPLC. The significance of differences in protein abundance was established using *p* ≤ 0.001 and |FC| ≥ 2 as thresholds (Table S5). Based on these criteria, 61 differentially regulated proteins (DRPs) were detected between *W. magna* cultivated in suspension and adhesion, of which 21 (34.4%) were down-regulated and 40 (65.6%) were up-regulated in adhesion compared with suspension cell growth (Table S5).

Among the 61 DRPs, 32 had a function assigned in the NCBI nr database, whilst 28 proteins had an uncharacterized function, including 5 hypothetical proteins, 21 predicted proteins and 2 ORFans. Most of the proteins (*n* = 52) had best hits with *Naegleria gruberi*, a free-living amoeba phylogenetically close to *W. magna* (Table S5). The proteins exhibiting the greatest differential abundance between the two conditions were proteins with unknown functions for amoebas in both suspension and adhesion culture (Table S5).

**Figure 2:**
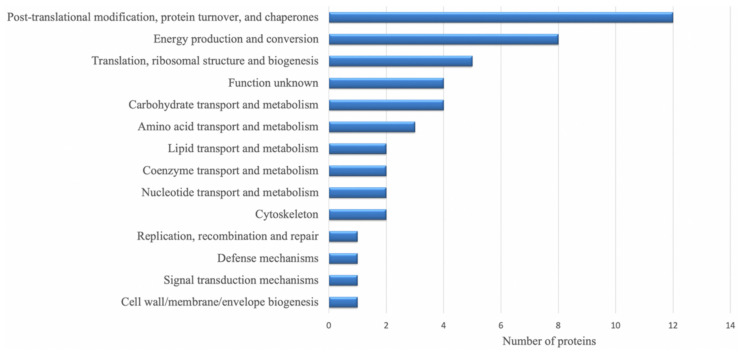
Representation of differently regulated proteins (DRPs) matching with a function in the Cluster of Orthologous Group of proteins (COG) database.

A total of 48 DRPs (78.7%) identified by proteomics analysis, were classified into 14 COG categories, among which “post-translational modification, protein turnover, chaperones” represented the largest group (group O, 12 DRPs), followed by “energy production and conversion” (group C, 8 DRPs) and “translation, ribosomal structure and biogenesis” (group J, 5 DRPs) (Figure 2, Table S5).

**Figure 3:**
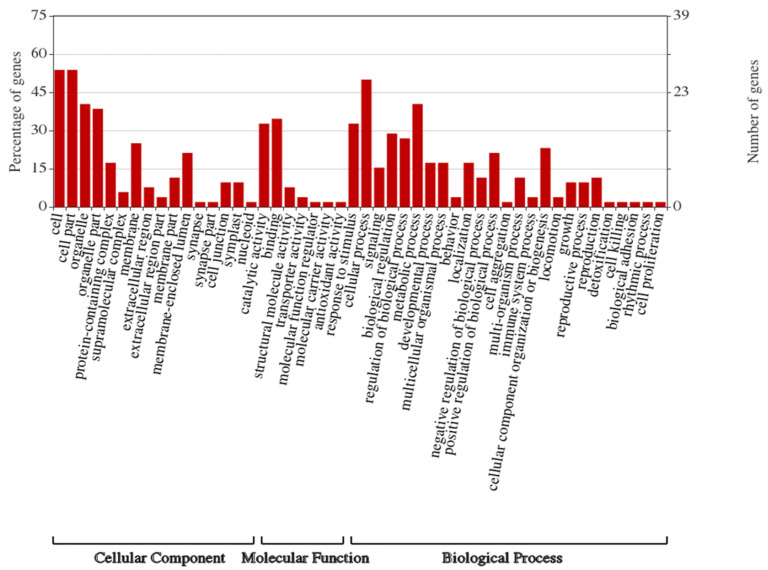
Gene Ontology (GO) distribution plotted by WEGO. The proteins were assigned to three main categories: biological process, molecular function and cellular component. The right-hand y-axis indicates the number of annotated proteins. The left-hand *y*-axis indicates the percentage of annotated proteins.

GO analysis was carried out to obtain fundamental function of genes and information on molecular functions, cellular components and biological processes. After mapping the DRPs against the GO database, we observed that 28 genes (45%) were assigned to GO terms. A same gene could be involved in different GO terms. Genes among the following classes: biological process (27), cellular process (26), metabolic process (21) and response to stimulus (17) were the most abundant. For molecular function (26), the dominant group was binding (18) followed by catalytic activity (17) and structure molecular activity (4). For the cellular components (28), the three most prevalent groups were cell (28), cell part (28) and organelle (21) (Figure 3).

**Figure 4:**
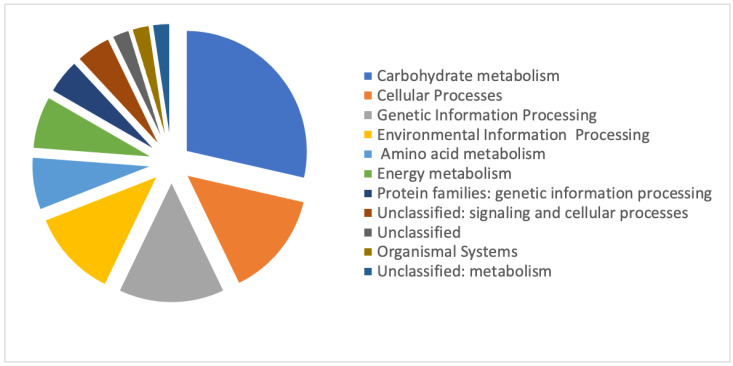
Representation of DRPs matching with a function in the Kyoto Encyclopedia of Genes and Genomes Pathway (KEGG) database. Each protein was classified in a KEGG category.

To investigate the biological functions and metabolic pathways of protein sequences, we performed an enrichment analysis with the KEGG pathway database. Among the 61 DRPs, 45 (72.6%) DRPs were mapped to 22 pathways in the KEGG database. The proteins involved in metabolic pathways were the most represented (*n* = 12; 27%), followed by those involved in cellular processes (13%) and genetic information processing (13%). KEGG enrichment indicated that most of these DRPs were involved in carbohydrate metabolism, such as citrate cycle and glycolysis/gluconeogenesis. We also identified some DRPs involved in “nucleotide metabolism” (*n* = 1; 2%) and “lipid metabolism” (*n* = 1; 2%) (Figure 4).

### 3.3. Identities of Differentially Regulated Proteins

Among the DRPs identified for the amoebas cultivated in adhesion, we found actin alpha, actin beta, tubulin, and profilin proteins (Table 1). These four proteins are related to the cytoskeleton organization. Of the 40 DRPs in adhesion cell growth, a high number (*n* = 13; 31%) were found related to metabolisms. Most of these proteins are involved in carbohydrate metabolism, including malate dehydrogenase, pyruvate dehydrogenase, glyceraldehyde 3-phosphate dehydrogenase, triose phosphate isomerase, or citrate synthase (Table 1). Furthermore, we observed an upregulation of 4 proteins involved in the nucleotide and amino acid pathway, such as glutamate dehydrogenase or cystathionine gamma-synthase (Table 1). In addition to the proteins related to metabolisms, the analysis revealed in adhesion cell growth a high abundance of proteins related to energy, signaling and redox regulation (thioredoxin) (Table 1). In a previous study, we reported the presence of proteins specifically related to defense mechanism in the proteome of *W. magna* cultivated in suspension [33]. In our differential proteomic analysis, we recovered some of these proteins related to the mechanism, including four cathepsin proteins involved in catabolism and lysosomal protease. These cathepsins were the only proteins related to the defense mechanisms that were differentially regulated according to the culture method (Table 1). For the cells cultivated in suspension, we reported an upregulation in suspension cell growth of proteins related to stressful conditions such as the heat shock condition (*n* = 4, heat shock proteins) (Table 2). In addition, we observed the over-regulation of two proteins playing a role in the membrane structure and four proteins involved in metabolism pathways, including lipid (5 long-chain acyl-CoA synthetase and alcohol dehydrogenase) and amino acid metabolisms (maleylacetoacetate isomerase and 5-methyltetrahydropteroyltriglutamate--homocysteine methyltransferase) (Table 2). Finally, two proteins (large subunit ribosomal protein L7/L12 and Ran GTPase binding) related to genetic information were found to be over-regulated and three were down-regulated in suspension cell growth (large subunit ribosomal protein L24e, large subunit ribosomal protein L23e and RRM domain-containing protein) (Table 1 and Table 2). To complete the differential proteomic analysis, we also investigated the expression levels by qRT-PCR of 10 selected genes encoding DRPs. The transcription levels of these 10 mRNAs were correlated with the results of differential proteomics analysis (Figure S1 and Table S3).

### 3.4. Analysis of Lipid Classes

First, we described the lipid classes of the amoeba cultivated according to two different conditions. The LC/MS analysis reported overall similar lipid classes profiles, including apolar lipids, free fatty acids, phospholipids, sphingosines and lysophospholipids (Table S6). The peak areas for each lipid class were comparable between the culture conditions and these results were confirmed for both positive and negative electrospray ionization modes used in the study (Table S6). According to the LC/MS results, the lipid classes that exhibited the greatest signal were phospholipids, precisely phosphatidylcholines (PCs) and phosphatidylethanolamines (PEs) (Table S6). Nevertheless, the LC/MS analysis of lipid classes displayed different chromatogram peak profiles for cells cultivated under two different conditions (suspension and adhesion). Chromatogram profiles were comparable between samples and injection replicates. This fact suggests the presence of different chemical structures between the two conditions within the same lipid classes. The multivariate analysis of the raw data confirmed the structure differences by PCA for the positive and negative ionization modes independently (Table S7). We then attempted to identify the discriminating markers and their belonging class. The extracted ion chromatograms were compared to known standards and the exact masses computed with the LipidMAPS database (Table S7). The results are summarized in Table 3. These results showed fewer markers in the case of cells grown in suspension. The adhesion culture indicated several lysophospholipid markers and a greater diversity of glycolipid or phospholipid markers. In a second time, we investigated the composition of total fatty acids (free and included in complex lipids, such as phospholipids or acylglycerols). Comparative analysis of total fatty acids profiles revealed similar classes of structure, namely saturated, unsaturated and branched carbon chains (Table S8). Yet, we found a higher abundance of longer carbon backbones for the amoebas cultivated in the adhesion mode (Table S8).

## 4. Discussion

For the first time, the behavior of a FLA is compared according to two culture modes using multi-omics approaches.

A previous transcriptomics and proteomics study showed that *W. magna* C2c Maky cultivated in a bioreactor had the weapons to deal with bacterial attacks in the environment [33]. Indeed, we detected production of several proteins involved in the mechanism defenses of eukaryotic organisms against foreign microbes. Among the proteins detected in the DRP study, we observed that the essential proteins associated with the defense mechanisms were not differentially regulated and were therefore probably not impacted by the culture method.

However, we reported some differences between the two culture modes for the cell growth. Indeed, the protist seems to promote the metabolism of carbohydrate when cultivated in adhesion. This finding was correlated with an over-regulation of proteins related to energy. Culture media were similar for each cell growth, but fetal bovine serum (FBS) was removed from the amoebal culture in suspension to reduce the cost of large-scale cultivation. We can suggest that the proteins and growth factors contained in the FBS could improve the assimilation of glucose by *W. magna* in adhesion culture. Therefore, a source of carbohydrate (other than glucose) could be added to the media to provide more energy at the cells cultivated in suspension. Glucose oxidation is an important energy source for cells; however, this process generates the formation of reactive oxygen species (ROS) extremely harmful to the cells. To mediate the oxidative stress, thioredoxin proteins were over-regulated for *W. magna* cultivated in adhesion [62,63]. The energy provided by carbohydrate metabolism is notably essential to ensure amoeboid mobility and maintaining the cytoskeleton. Among the DRPs, we observed the over-regulation of proteins related to the cytoskeleton structure. Amoebas migration is initiated by the polymerization of actin that allows the formation of the organelle indispensable for amoeboid movement [64]. The over-regulation of these proteins revealed a dynamic activity of *W. magna* cultured in adhesion. In addition, these results were correlated with the large size and quantity of amoeba pseudopods in adhesion culture.

When *W. magna* was cultured in bioreactor, we observed over-regulation of long-chain acyl- coenzyme A (CoA) synthetase and alcohol dehydrogenase which are proteins involved in the lipid metabolism pathway. Alcohol dehydrogenase is an enzyme regulating the biosynthetic pathway of glycerol [65]. This carbon source is converted into intermediate metabolites used for the lipid biosynthesis, including the formation of fatty acids activated through acyl-CoA [66,67]. In addition, we found an over-regulation of homocysteine methyltransferase protein, which is an enzyme implicated in methionine formation [68]. This enzyme is also known to be involved in the metabolism of PCs, a class of over-represented lipids for the amoebas cultivated in bioreactor (in suspension). The use of choline or homocysteine, which are precursors of methionine, could be beneficial to improve the culture of amoebas in bioreactors. Otherwise, the stress of cells cultivated in suspension was revealed by the over-regulation of heat-shock proteins. A drop-in culture temperature could thus allow the production of less stressed cells.

An investigation on total lipid classes was achieved to complement the proteomics analysis. Our survey has shown that amoebas are composed of a similar set of lipid classes regardless of the culture mode. Interestingly, we found that phospholipids were the most represented lipids for *W. magna* cultivated in the two culture modes. However, we pointed out specific lipid markers according to the culture condition used. Thus, growth cell in adhesion showed the presence of specific glycolipid markers that could be related to the physical state of the amoebas and that could be involved in cell-to-cell interactions [69,70]. Noteworthy, glycosphingolipids are known to be involved in the cell-to-cell communication [71,72]. In adhesion, *W. magna* consumed a large amount of carbohydrates, which could be correlated with a high proportion of glycosylated lipids. Lysophospholipids have been reported to be involved in the membrane shaping process and bending, but also as mediators in signaling cascades for migration or cell-to-cell attachment [73,74]. Their increase during adhesion cell growth compared to suspension could therefore be linked to these processes. *W. magna* cells in adhesion compared to the suspension condition exhibited the formation of phosphatidic acid structures, which are usual minor components of the cell membrane. Speranza et al. reported the interaction of PAs with actin-related proteins in order to promote actin polymerization [75]. The higher contents of phosphatidylcholine lipid markers that we observed in suspension culture condition could be related to these proteomics results.

## 5. Conclusions

In conclusion, the study allowed to improve knowledge on the understudied amoebic field. This survey provides new insights into the mechanisms used by *W. magna* to adapt to different culture conditions. Indeed, *W. magna* regulated its protein and lipid metabolism to grow in different culture conditions. Therefore, multi-omics approaches are essential tools to study the molecular responses and biology of FLAs.

## Figures and Tables

**Table 1:** *Willaertia magna* proteins up-regulated in adhesion and down-regulated in suspension culture.

Protein Sequence Accession of *W. magna*	Anova (p)	Fold Change	Protein Function
protein 17463	1.45 × 10^−14^	410.39	predicted protein
protein 8919	4.00 × 10^−8^	54.12	uncharacterized protein
protein 2287	5.55 × 10^−16^	44.36	cathepsin B-like protein
protein 8084	5.68 × 10^−14^	7.2	malate dehydrogenase
protein 15939	4.35 × 10^−13^	6.48	Ras GTP-binding protein RhoL
protein 4754	1.81 × 10^−9^	5.96	Hemerythrin-like protein
protein 15039	1.81 × 10^−9^	5.96	Hemerythrin-like protein
protein 17074	2.62 × 10^−6^	4.88	Unknown function (ORFan)
protein 15365	1.43 × 10^−12^	4.8	predicted protein
protein 13743	3.72 × 10^−6^	4.7	ubiquinone biosynthesis protein
protein 8031	3.75 × 10^−8^	4.11	cathepsin B-like protein
protein 4072	1.84 × 10^−8^	4.02	glyceraldehyde-3-phosphate dehydrogenase
protein 17615	6.17 × 10^−6^	3.95	V-type H+-transporting ATPase subunit F
protein 2899	1.20 × 10^−9^	3.24	beta-tubulin
protein 3274	7.59 × 10^−12^	3.16	adenylate kinase
protein 7439	4.31 × 10^−8^	2.99	triosephosphate isomerase
protein 7923	2.67 × 10^−4^	2.82	protein domain specific binding
protein 12867	1.20 × 10^−5^	2.78	arsonoacetate metabolic process
protein 2947	1.27 × 10^−11^	2.65	3-oxoacid CoA-transferase activity
protein 8114	1.69 × 10^−3^	2.47	cathepsin B-like protein
protein 7780	4.69 × 10^−4^	2.41	pyruvate dehydrogenase
protein 5985	2.67 × 10^−6^	2.4	alpha-actinin
protein 3614	1.27 × 10^−10^	2.39	malate dehydrogenase
protein 885	2.48 × 10^−7^	2.39	RRM domain-containing protein
protein 13554	2.66 × 10^−10^	2.39	glutamate dehydrogenase
protein 3571	5.22 × 10^−5^	2.38	Profilin
protein 8014	5.79 × 10^−8^	2.25	phosphoenolpyruvate carboxykinase
protein 6333	3.93 × 10^−3^	2.21	predicted protein
protein 3628	1.13 × 10^−5^	2.2	cathepsin D
protein 16124	1.07 × 10^−9^	2.19	ADP/ATP translocase 1 domain protein
protein 8489	3.94 × 10^−6^	2.19	actin beta/gamma 1
protein 11280	1.15 × 10^−3^	2.14	60S ribosomal protein L23
protein 4486	6. × 10^−11^	2.13	cystathionine gamma-lyase
protein 2738	5.05 × 10^−5^	2.12	cathepsin B
protein 8503	3.55 × 10^−10^	2.11	ATP synthase F1 subunit alpha
protein 7955	3.57 × 10^−6^	2.1	large subunit ribosomal protein L24e
protein 4073	7.58 × 10^−9^	2.05	fructose-bisphosphate aldolase
protein 15452	3.15 × 10^−4^	2.02	malate metabolic process
protein 2384	6.51 × 10^−9^	2.02	mitochondrial citrate synthetase
protein 18191	2.88 × 10^−9^	2.01	thioredoxin

**Table 2:** *Willaertia magna* proteins up-regulated in suspension and down-regulated in adhesion culture.

Protein Sequence Accession of *W. magna*	Anova (p)	Fold Change	Protein Function
protein 5557	1.63 × 10^−10^	Infinity	Unknown protein (ORFan)
protein 3984	4.59 × 10^−12^	15.52	large subunit ribosomal protein L7/L12
protein 2294	3.31 × 10^−13^	11.34	metE; 5-methyltetrahydropteroyltriglutamate–homocysteine methyltransferase
protein 16136	8.95 × 10^−14^	5.98	membrane protein
protein 2157	5.28 × 10^−4^	4.12	ribosomal protein L7/L12, putative
protein 10142	4.22 × 10^−14^	3.43	HSP-20 domain-containing protein
protein 18401	8.68 × 10^−7^	3.41	nuclear transport factor 2
protein 18200	5.01 × 10^−6^	2.84	maleylacetoacetate isomerase
protein 2831	8.88 × 10^−16^	2.68	HSP20 family protein
protein 11490	2.19 × 10^−4^	2.59	predicted protein
protein 2849	3.97 × 10^−8^	2.49	predicted protein
protein 8080	3.66 × 10^−6^	2.46	long-chain acyl-CoA synthetase
protein 11879	7.72 × 10^−5^	2.3	hypothetical protein
protein 15002	2.04 × 10^−10^	2.21	membrane protein
protein 16271	6.39 × 10^−12^	2.18	heat shock 70kDa protein 5
protein 3173	6.20 × 10^−6^	2.18	mitochondrial chaperonin hsp10
protein 14923	5.72 × 10^−7^	2.15	predicted protein
protein 13231	5.57 × 10^−7^	2.15	sugar phosphate isomerase/epimerase
protein 13228	3.71 × 10^−3^	2.12	predicted protein
protein 6869	5.31 × 10^−7^	2.07	alcohol dehydrogenase
protein 2046	7.87 × 10^−5^	2.02	catalase

**Table 3:** Identification of lipid markers related to different culture modes.

Culture Condition	Suspension	Adhesion
Positive ionization	4 apolar lipids (1 Ceramide, 3 Acylglycerols) 4 Glyco lipids (Glycosyldiacylglycerols) 11 phospholipids (6 PCs, 5 unknown)	2 apolar lipids (Acylglycerols) 5 Glyco lipids (3 Hex Sphingosine, 2 Glycosyldiacylglycerols) 7 Phospholipids (3 PCs, 1 phosphatidyl serine, 3 unknown) 7 Lysophospholipids
Negative ionization	2 apolar lipids (Ceramides) 8 Free Fatty Acids (7 identified) 6 phospholipids (1 PE, 5 PC) 1 lysophospholipid	5 apolar lipid (1 Ceramide, 1 Wax Ester, 1 Coenzyme A, 2 unknown) 2 Glyco lipids (1 Ceramide, 1 Glycosyldiacylglycerol) 13 Free Fatty Acids (6 identified) 6 Phospholipids (1 phosphatidylinositol, 1 phosphatidic acid, 4 PEs) 14 Lysophospholipids

## Supplementary Materials

The following are available online at https://www.mdpi.com/2076-2607/8/11/1791/s1. Supplementary data (word file) contain Figure S1 and Table S3, Figure S1: comparison between qRT-PCR and proteomic results of abundance proteins, Table S1: data obtained from protein extractions of *W. magna* cultivated in different culture conditions, Table S2: The results obtained from proteomics analysis, Table S3: primers used for the quantification of mRNAs expression level by qRT-PCR, Table S4: measuring the length and width of the amoebas cultivated under two different conditions, Table S5: identification and annotation of the DRPs, Table S6: data of lipid classes profiles performed by LC/MS analysis, Table S7: identification of lipid markers related to different culture modes, Table S8: analysis of total fatty acids profiles.
