# Supplementary material for: Proteomics and Lipidomics Investigations to Decipher the Behavior of Willaertia magna C2c Maky According to Different Culture Modes
Source: Microorganisms. 2020 Nov 16;8(11):1791. doi: 10.3390/microorganisms8111791 (PMC7696429; doi:10.3390/microorganisms8111791)

**Title: Proteomics and lipidomics investigation to decipher the behavior of *Willaertia magna* C2c Maky according to the different culture methods.**

**Authors:** Issam Hasni^1,2,3^, Nicholas Armstrong^1,3^, Philippe Decloquement^1,3^, Said Azza^1,3^, Anthony Fontanini^1,3^, Olivier Abbe^2^, Amina Cherif Louazani ^1,3^, Sandrine Demaneche^2^, Eric Chabrière^1,3^, Philippe Colson^1,3^ and Bernard La Scola^1,3^*

**Supplementary data:**

**Table S3 Primer used for the quantification of mRNAs expression level by qRT-PCR**

|  | **Forward (5'->3')**  **Reverse (5'->3')** | | **TM (C°)** | | **size of amplicon in base pair (bp)** |
| --- | --- | --- | --- | --- | --- |
| Gene 16136 (protein1) | aaccggtatttgcgttccag | | 59 | 106bp | |
|  | tgagttaaagtctggggttggg | 60 | |  | |
| Gene 11879 (protein2) | caagcacccaaagctcttactg | 60 | | 148bp | |
|  | cgtgtttttcaccacattcagc | 59 | |  | |
| Gene 8080 (protein3) | aactgaaacagctgctggtg | 60 | | 142bp | |
|  | tggtggattgtcagttgcac | 60 | |  | |
| Gene 2899 (protein4) | tgctaatccaggtggtttaggg | 60 | | 143bp | |
|  | ttcctccacctgacattgtagc | 60 | |  | |
| Gene 2831 (protein5) | tctttggtggtttcccatctg | 59 | | 124bp | |
|  | tctttgacatctacagctggttg | 59 | |  | |
| Gene 8080 (protein6) | agagaagtggaaataaaacaacg | 60 | | 230bp | |
|  | tgacaagtaattgataattgatct | 60 | |  | |
| Gene 2831 (protein7) | gatgcacaattaaacggtaccg | 58 | | 150bp | |
|  | tgtatctaaatgtccaccacgtg | 59 | |  | |
| Gene 2899 (protein8) | aattgcacactcccttggtg | 59 | | 149bp | |
|  | tgtaaggttcgaccacggtatc | 60 | |  | |
| Gene 5985 (protein9) | tctgaagacgaactttctgcaag | 60 | | 112bp | |
|  | aaccatcttgccatgaaccc | 59 | |  | |
| Gene 3571(protein10) | gccagttactgcacaagaagc | 60 | | 146bp | |
|  | ctgcatttgcgaaaccttgg | 59 | |  | |
| Gene 4130 (histone) | aggtggtgaaaaagatgatgacg | 60 | | 143bp | |
|  | tggacattgccttgttggag | 58 | |  | |

**Figure S1:** Comparison between qRT-PCR and proteomic results of abundance proteins. X-axis represented protein identification, the blue column represented proteomic results, the green column represented qRT-PCR results; Y-axis represented the abundance fold change of DEPs and mRNAs. The proteins (1,2,3,4 and 5) are up regulated in suspension and down regulated in adhesion. The proteins (6,7, 8, 9 and 10) are upregulated in adhesion and down regulated in suspension.


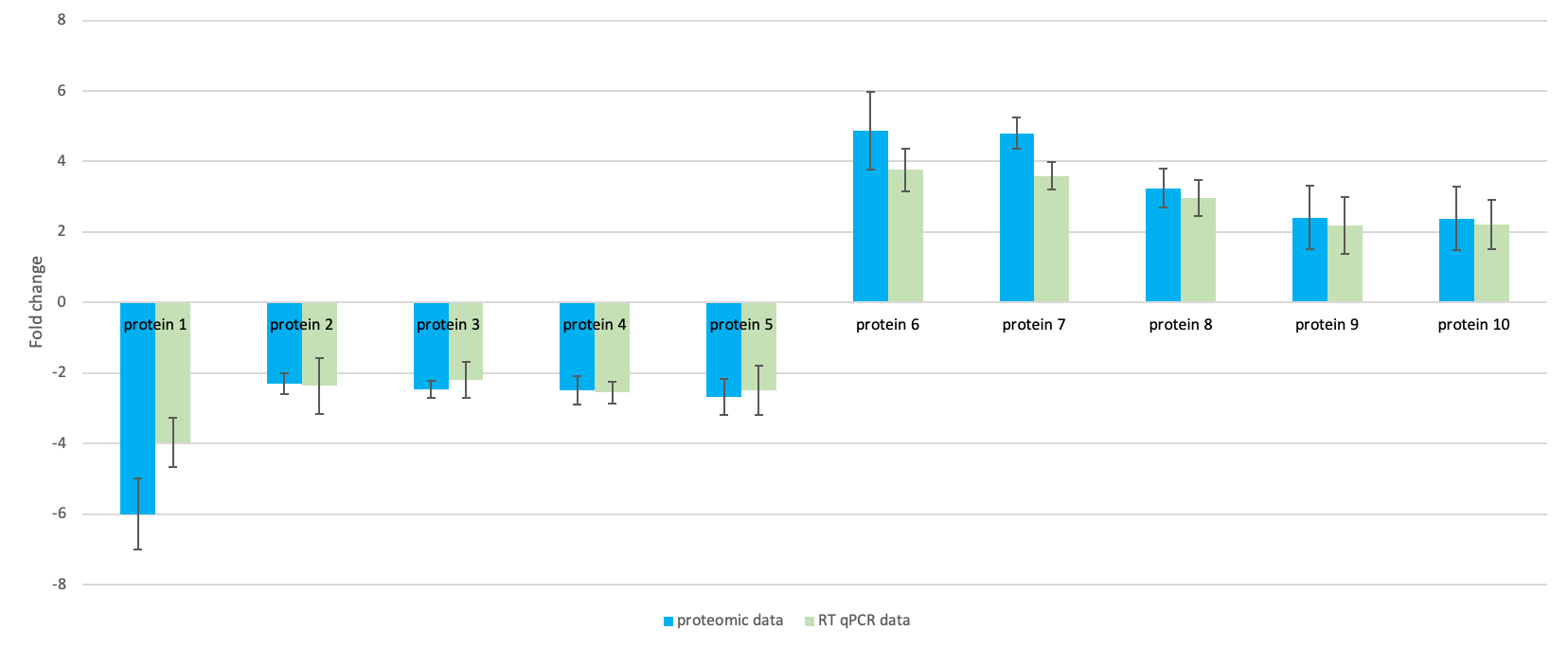

Supplement: Supplementary file 1 [file microorganisms-08-01791-s001.zip › Supplementary data and Table S3.docx]
